# Image-Derived Traits Related to Mid-Season Growth Performance of Maize Under Nitrogen and Water Stress

**DOI:** 10.3389/fpls.2019.00814

**Published:** 2019-06-26

**Authors:** Dejan Dodig, Sofija Božinović, Ana Nikolić, Miroslav Zorić, Jelena Vančetović, Dragana Ignjatović-Micić, Nenad Delić, Kathleen Weigelt-Fischer, Astrid Junker, Thomas Altmann

**Affiliations:** ^1^Department for Research and Development, Maize Research Institute Zemun Polje, Belgrade, Serbia; ^2^Department for Maize, Institute of Field and Vegetable Crops, Novi Sad, Serbia; ^3^Department of Molecular Genetics, Leibniz Institute of Plant Genetics and Crop Plant Research (IPK), Gatersleben, Germany

**Keywords:** maize genotypes, high-throughput phenotyping, vegetative biomass, nitrogen deficiency, water stress, variable selection, stress indices

## Abstract

Phenotypic measurements under controlled cultivation conditions are essential to gain a mechanistic understanding of plant responses to environmental impacts and thus for knowledge-based improvement of their performance under natural field conditions. Twenty maize inbred lines (ILs) were phenotyped in response to two levels of water and nitrogen supply (control and stress) and combined nitrogen and water deficit. Over a course of 5 weeks (from about 4-leaf stage to the beginning of the reproductive stage), maize phenology and growth were monitored by using a high-throughput phenotyping platform for daily acquisition of images in different spectral ranges. The focus of the present study is on the measurements taken at the time of maximum water stress (for traits that reflect plant physiological properties) and at the end of the experiment (for traits that reflect plant architectural and biomass-related traits). Twenty-five phenotypic traits extracted from the digital image data that support biological interpretation of plant growth were selected for their predictive value for mid-season shoot biomass accumulation. Measured fresh and dry weights after harvest were used to calculate various indices (water-use efficiency, physiological nitrogen-use efficiency, specific plant weight) and to establish correlations with image-derived phenotypic features. Also, score indices based on dry weight were used to identify contrasting ILs in terms of productivity and tolerance to stress, and their means for image-derived and manually measured traits were compared. Color-related traits appear to be indicative of plant performance and photosystem II operating efficiency might be an importance physiological parameter of biomass accumulation, particularly under severe stress conditions. Also, genotypes showing greater leaf area may be better adapted to abiotic stress conditions.

## Introduction

Nitrogen and water, separately or in combination, are two of the most critical factors in maize production worldwide. Nitrogen is a major growth and yield-determining plant nutrient and its major uptake by maize plants is often referred to start at the stage of six fully expanded leaves when rapid growth begins, and to continue into the reproductive stage. Twelve and seventeen percent yield reduction, respectively, has been reported to be attributed to irreversible effects of delayed nitrogen application at the 6- and 10-leaf stages (Binder et al., 2000; Walsh et al., 2012). Nitrogen accumulated in vegetative organs prior to silking is remobilized by plants to grains as new nitrogen taken up during reproductive development is not sufficient for maize grain filling (Mueller and Vyn, 2016). Maize water requirement is highest in the reproductive stage (Kranz et al., 2008), however water shortage during vegetative growth can also significantly reduce grain yield. Short-term water deficits during rapid vegetative growth caused up to 40% grain yield losses which was explained by a decline in plant extension growth and a reduction of leaf size (Çakir, 2004). The responses of plants to a combination of water and nitrogen stress may even cause further effects beyond the individual impacts, and hence cannot be directly extrapolated from conclusions obtained from the different stresses applied individually (Humbert et al., 2013). Several studies showed that it was possible to improve maize germplasm for simultaneous expression of tolerance to mid-season drought and nitrogen stress through recurrent selection (Bänziger et al., 2002; Zaidi et al., 2004).

Many efforts have been made to improve the mechanistic understanding of plants tolerance to abiotic stresses. The development of high-throughput phenotyping platforms, with a variety of imaging methodologies, provide a new prospect for dissecting complex plant traits such as stress tolerance into functionally relevant components (Tardieu and Tuberosa, 2010; Chen et al., 2014; Li et al., 2014; Rahaman et al., 2015). Although high-throughput automated imaging is not without its limitations (Li et al., 2014) this technology is becoming more advanced and popular, due to the capability to non-destructively capture various traits at regular time intervals throughout the life cycle of the plant (Rahaman et al., 2015; Muraya et al., 2017). Numerous studies in different crop species including maize reported high correlations between image-derived traits and traits recorded by traditional metrics thus validating digital imaging as a reliable tool for phenotyping (Nagel et al., 2012; Honsdorf et al., 2014; Humplík et al., 2015; Neilson et al., 2015; Neumann et al., 2015; Arend et al., 2016; Ge et al., 2016). Moreover, Zhang et al. (2017) suggested that projected plant area acquired from side imaging can replace dry weight in quantitave trait locus (QTL) analysis in maize according to the matches of QTLs affecting dry weight and projected plant area. Recent studies suggested that high-throughput phenotyping offers a powerful entry into dissecting genetic components underlying plant biomass accumulation (Muraya et al., 2017; Zhang et al., 2017; Chen et al., 2018).

The identification of stress resistance of different genotypes is an important goal in crop breeding programs. A stress resistant genotype can be defined as one which gives a significantly higher yield than average under conditions where crop resources availability are limited by some aspect of the environment (Quarrie et al., 1999). To differentiate stress adaptation levels of genotypes, several selection indices have been suggested on the basis of yield or biomass performance of a given genotype under stress and non-stress conditions (Rosielle and Hamblin, 1981; Fernandez, 1992) or in comparison with the average yield (Fischer and Maurer, 1978; Fernandez, 1992). More recently, to overcome limitations of using various indices *per se* Thiry et al. (2016) proposed a new method based on a scoring scale involving a combination of previously developed stress indices. This new method offers a simple way to identify best or worst crop genotypes within a population, in terms of resilience of stress and production capacity. In addition, identified contrasting genotypes are essential prerequisites for investigations of the possible roles of specific traits in genotypic responses to stress conditions.

Here, we used an automated high-throughput plant phenotyping facilities to investigate maize morpho-physiological responses to optimal, limited nitrogen supply, limited water supply and combined nitrogen and water stress during vegetative growth (before tasseling). In this study we focused on the measurements done at the phase of maximal water stress and at the end of the cultivation period with two main objectives: (i) to identify reliable and useful image-based traits for mid-season biomass accumulation in each treatment and (ii) to identify contrasting genotypes in a terms of biomass productivity for each stress type with image-derived and manually measured traits contributing to stress tolerance.

## Materials and Methods

### Plant Material

Twenty temperate maize inbred lines (ILs) were selected for the experiment. The selection of the 20 ILs was done so as to represent a set of public and commercial lines with variation in terms of tolerance to abiotic (mainly drought) stresses. ILs B73 (IL1), A632 (IL2), and Mo17 (IL3) have been chosen as some of the most famous representatives of public sector inbreds. These historical inbreds are widely recognized as sensitive to drought. V-273 (IL4) is a commercial line developed at the Maize Research Institute Zemun Polje (MRIZP), Serbia and represents a prolific (multi-ear) version of B73 inbred. The IL4 is still being used as a female parent in several MRIZP hybrids and under heat and/or drought stress firing of leaves and lower yield potential may be expected. V-395/31 (IL5) is a public IL that has been used as a female parent in several MRIZP hybrids grown in Serbia during the 1970s. It has been developed from an old Yugoslavian population Vukovar and according to our knowledge is susceptible to drought. L 375/25-7 (IL6), L 325/75-2 (IL7), and L 335/99 (IL18) are most recently developed MRIZP elite ILs that are being used both as female and male parental components in several widely grown hybrids in Serbia and abroad. They show good general combining abilities and are known to be tolerant to drought during critical growth stages for water requirement. Genotypes TVA1415-1 (IL8), 727574 (IL9), TVA912-1 (IL10), PZS61 (IL11), ČK674/78-2 (IL12), TVA810-1 (IL13), RC109 (IL14), Vir44 PEP (IL15), UČ23 (IL16), S49 (IL17), TVA1736-1 (IL19), and TVA303-1 (IL20) are introduced ILs from the MRIZP Gene bank collection used for broadening genetic diversity of the elite MRIZP breeding material. They belong to a drought tolerant mini core collection that was established after screening of entire MRIZP Gene bank accessions for drought tolerance for 2 years (Vančetovć et al., 2010; Babić et al., 2011). Additionally, unique SNP mutations in the *ZmMYBE1* gene, involved in the regulation of growth rate, plant height and photoperiod in maize (Jia et al., 2009), were found in inbreds IL9, IL11, IL12, IL13, and IL14 (Assenov et al., 2013). Further information on ILs used in this study such as maturity group, developmental or collection origin, and the germplasm pools they belong to is given in Table 1. All seeds used in this study were multiplied at MRIZP in a single year under non-stress conditions.

**Table 1:** List of the inbred lines used in this study and information of their maturity groups, the developmental origins, the sector/ownership and the germplasm pools they belong to.

Name	Code	Maturity group	Gene pool	Sector	Origin
B-73	IL1	Late	Dent	Public	United States
A-632	IL2	Intermediate	Dent	Public	United States
Mo-17	IL3	Late	Dent	Public	United States
V-273	IL4	Late	Semi dent	Private	Serbia
V-395/31	IL5	Late	Dent	Public	Yugoslavia
L 375/25-7	IL6	Intermediate	Dent	Private	Serbia
L 325/75-2	IL7	Late	Dent	Private	Serbia
TVA1415-1	IL8	Early	Dent	Public	Czechoslovakia^†^
727574	IL9	Intermediate	Semi dent	Public	United States^†^
TVA912-1	IL10	Intermediate	Flint	Public	Yugoslavia^†^
PZS61	IL11	Early	Dent	Public	USSR^†^
ČK674/78-2	IL12	Intermediate	Semi flint	Public	USSR^†^
TVA810-1	IL13	Intermediate	Dent	Public	Czechoslovakia^†^
RC109	IL14	Intermediate	Semi dent	Public	Czechoslovakia^†^
Vir44 PEP	IL15	Intermediate	Dent	Public	USSR^†^
UÈ23	IL16	Intermediate	Dent	Public	USSR^†^
S49	IL17	Early	Flint	Public	Poland^†^
L-335/99	IL18	Late	Flint	Private	Serbia
TVA1736-1	IL19	Intermediate	Dent	Public	Czechoslovakia^†^
TVA303-1	IL20	Early	Dent	Public	Czechoslovakia^†^

*†Indicates the MRIZP Gene bank accessions for which exact country of development could not be determined so country of collection was listed instead. Based on our knowledge or as reported in past studies (see section “Materials and Methods” for more details) the studied genotypes can be classified as susceptible (ILs 1-5) or tolerant (ILs 6-20).*

### Experimental Set-Up and Phenotyping

The experiment was performed in a climate controlled glasshouse of the Leibniz Institute of Plant Genetics and Crop Plant Research (IPK), Gatersleben, Germany. The automated phenotyping platform for large plants (Junker et al., 2015) was used to characterize 20 diverse maize ILs for their responses to nitrogen deficiency (N) and water stress (W), as well as combined nitrogen and water stress (N + W) imposed mainly during the vegetative developmental phase. Control treatment (C) involved adequate water and nitrogen supply. In each treatment, eight plants per IL were tested, which resulted in a total of 640 plants. To ensure phenological synchronization across ILs at the targeted stage when nitrogen and water stress were to be imposed (starting from around 6-leaf stage), a pre-study was performed to determine the phenology of ILs in greenhouse conditions. Information on the number of days to 6-leaf stage was used as covariate adjustment to group genotypes into subsets of similar phenology (early, intermediate and late, Table 1) for sowing at different times. Sowing of seeds was first done for late ILs, followed by ILs belong to intermediate and early vegetative groups at 3-day intervals. Sixty-four seeds per each IL were sown in small pots (one plant per pot) for germination and seedlings pre-culture.

On 17, 14, and 11 days after sowing of late, intermediate, and early vegetative groups, respectively, 32 visually uniform plants per IL were transplanted into larger pots. At the time of transplanting, plants had reached approximately the 4-leaf developmental stage. After transplanting, the pots were transferred into IPK’s automated plant phenotyping (IPK-APP) system for large plants placed in climate controlled glasshouse and ILs were grown for further 35 days more. At the time of stresses impose plants approximately reached 6- to 7-leaf developmental stage. At the time of maximum water stress plants were about from 9- to 11-leaf developmental stage in all treatments. At the end of experiment plants had approximately reached the 11- to 14- (in W and N + W) leaf developmental stage and 12-to 16- (in C and N) leaf developmental stage (see Supplementary Table 1 for leaf stage comparison between the ILs). Briefly, each genotype was replicated 8 times per treatment, with replicates arranged in blocks of two plants (each in an individual pot) and placed onto one carrier on a conveyor system throughout the glasshouse compartment for joint movement, imaging, watering and fertilizing. The conveyor system consists of 12 lanes each storing 33 carriers. To avoid position effects, carriers were shuffled lane-wise 2–3 times per week. Carriers were moved to three consecutive imaging boxes for visible imaging (VIS, 390–750 nm), fluorescence imaging (FLUO, excitation: 400–500 nm, emission: 520–750 nm) and near infrared imaging (NIR, 1450–1550 nm), using in each case top view and side view CCD cameras (with 22°, 45°, 112°, and 135° side views). Additionally, during the experiment the system was upgraded with a FluorCam device (Photon Systems Instruments, Brno, Czechia) for kinetic chlorophyll fluorescence analyses (Tschiersch et al., 2017). The system also incorporates an automated weighing and watering unit for quantified delivery of both water and nutrient solutions to the plants throughout growth/measurement cycles. More details on the IPK-APP system and image acquisitions are given in Junker et al. (2015).

### Growth Conditions

During pre-cultivation, plants were grown in small 9 cm diameter pots (one plant per pot) filled with IPK soil mixture composed of 40% (v/v) IPK self-made compost + 40% (v/v) substrate 2 (Klasmann-Deilmann GmbH, Geeste, Germany) + 20% (v/v) sand (for compost and substrate composition see Junker et al., 2015). After the seeds were sown, the pots were kept in a climatized glasshouse chamber and watering was performed manually to allow optimal germination and seedling establishment. Plants were transplanted and entered IPK-APP in 5.5 l pots filled with the IPK soil mixture mentioned above.

The temperature regime during the experiment was set to mimick Zemun Polje vegetative temperature which raised stepwise sequentially during the growth period starting with 20/15°C day/night during germination and pre-culture period, then 22/17°C day/night for 10 days and finally to 25/20°C day/night temperature for further 25 days. During the entire cultivation period relative air humidity was set to a minimum of 65% and the light period was set to 16 h (06:00–22:00 h). For supplemental illumination SonT Agro high pressure sodium lamps (Philips, Amsterdam, Netherlands) were used to achieve an average total illumination of approx. 350 μmol m^-2^ s^-1^ PAR.

Plants were fertilized once at the beginning of the experiment (at 3 days after transplanting (DAT 3)) with a 75 ml solution containing 0.1% Wuxal^®^ Super (8% [w/w] nitrogen, 8% [w/w] P_2_O_5_, 6% [w/w] K_2_O, and micronutrients, MANNA). To realize two nitrogen levels different fertilizer solutions were applied once per week in next 4 weeks (at DATs 8, 15, 22, and 29). For optimal nitrogen conditions (C and W) 50 ml of 0.5% Wuxal^®^ Super fertilizer solution per pot was added, while for reduced nitrogen conditions (N and N + W) 50 ml of 0.03% Fetrilon^®^1-Combi (micronutrients without nitrogen, BASF) and 725 mg of KH_2_PO_4_ have been applied per pot. In total, 35 mg of nitrogen per pot was applied in optimal nitrogen conditions and 15 mg of nitrogen per pot in reduced nitrogen conditions.

In the C and N treatments, pots were watered daily to a target weight corresponding to 75% soil field capacity (SFC) from transplanting to DAT 35 (Supplementary Figure 1). The method for SFC determination was described in Junker et al. (2015). All carriers were weighed every day and the reduction of weight from 1 day to the next was used to calculate the amount of water lost from the soil. The standard cultivation protocol for maize at IPK-APP includes the use of blue cover material (rubber mats) for facile top view image segmentation and reducing water evaporation from the soil. In W and N + W treatments water stress was initiated at DAT 9 by cumulative soil drying to 20% SFC (DAT 22) and then raised to 30% SFC and kept at this level till the end of the experiment (Supplementary Figure 1).

### Image-Derived Plant Traits

Plants were imaged daily starting from 2 days after transplanting (DAT 2) to the end of the experiment (DAT 35). The Integrated Analysis Platform (IAP) was used for image (pre-) processing and automated feature extraction (Klukas et al., 2014). The multi-sensor setups at IPK (VIS, FLUO, NIR, FluorCam) support the assessment of around 200 traits corresponding to plant architecture, plant colorization, plant water content, or levels of fluorophores, as well as efficiency of photosystem II. In this study we focus on 25 phenotypic traits (extracted from images of each individual carrier) selected (i) to support biological interpretation of plant growth, (ii) to belong to different trait categories (Supplementary Table 2) and (iii) to show significant genotypic and/or treatment effects (Supplementary Table 3).

Selected image-derived traits could be broadly classified into three categories: architectural (length, area, shape, structure), physiological (fluo-based and color-related traits) and biomass-related traits. Detailed information for image-based trait definitions and details of trait extraction are shown in Supplementary Table 2. We here refer to their names and codes which will be used through the manuscript: side area (PSA), top area (PTA), side compactness (SCom), top compactness (TCo), convex hull area (CHA), solidity (Sol), surface coverage (SCov), caliper length (CLe), roundess (Rnd), plant height (PHg), plant width (PWd), leaf count (LCn), leaf width (LWd), leaf length (LLn), estimated biovolume (EBv), fluorescence intensity (FI), photosystem II efficiency (PSII), yellow to green (Y2G), brown to green (R2G), red to green (R2G), red color value (RGB_r), green color value (RGB_g), blue color value (RGB_b), Lab color a (Lab_a), and Lab color b (Lab_b). Although images from all standard modules (visible, fluorescence, and near-infrared) were available, we mainly used VIS images for selected traits. The static bulk fluorescence (IF) value was obtained from FLUO imaging (Junker et al., 2015) and pulsed amplitude modulated fluorescence parameters (PSII) from the FluorCam (Tschiersch et al., 2017) modules, respectively. Traits were derived from top or side view (averaged across different angles), or combined, as in a case of EBv [calculated as a volume from side and top view areas (Klukas et al., 2014), and can be used as a proxy for estimated biomass].

In this study we focused on the measurements done at the end of the experiment (DAT 35) for architectural and biomass-related traits. However, color-related traits were evaluated at the time of maximum water stress (DAT 22) as a previous study in barley (Neumann et al., 2015) showed that values of color-related traits were more different compared to that of the control plants during the water stress period than after re-watering. Obtained color-related traits data in this study also showed higher distinction among treatments at DAT 22 (when SFC was 20%) compared to DAT 35 when SFC was 30% (data not shown). The FluorCam device used for photosystem II operating efficiency (PSII) measurement became available at an advanced state of the stress treatment and was used once at DAT 23 (before watering applied that day, see Supplementary Figure 1).

### Manual Measurements of Traits and Indices

An overview of measured trait/indice definitions and methods of their extraction are given in Supplementary Table 2. A day before the end of the experiment (DAT 34) relative water content (RWC) in the youngest fully expanded leaf of each plant was determined. At the end of experiment (DAT 35) plants were removed at the soil level for biomass fresh- (BFw) and dry-weight (BDw) as well as chemical analyses (relative carbon concentration, CC and relative nitrogen concentration, NC in dry matter). Only data for NC (%) was shown, as for CC there were no significant differences between genotypes and treatments (ranged 39.2–43.6% across genotypes and treatments). Stress- and biomass-related indices such as specific plant weight (SPW), water use efficiency (WUE), physiological nitrogen use efficiency (PNUE), resilience capacity index (RCI) and production capacity index (PCI) were calculated based on BDw. Manual measurements were later used to establish correlations with image-based phenotypes.

### Identifying Contrasting Genotypes in Terms of Biomass Production Under Stress

To identify the degree of stress tolerance of different ILs used in this study we applied the screening method recently proposed by Thiry et al. (2016). Briefly, this new approach relies on the introducing of simple 10-grading (scoring) assesment for five stress indices previoulsly developed to evaluate drought adaptation: stress susceptibility index – SSI (Fischer and Maurer, 1978), stress tolerance index – TOL (Rosielle and Hamblin, 1981), mean productivity index – MP (Rosielle and Hamblin, 1981), geometric mean productivity – GMP (Fernandez, 1992) and stress tolerance index – STI (Fernandez, 1992). The calculations of stress indices are based on grain yield (in present case on a biomass dry matter basis) *per se* under stress and non-stress conditions. Once obtained, score indices have been classified within two new scales called RCI and PCI. New indices RCI (average indice score of SSI and TOL) and PCI (average indice score of MP, GMP and STI) were used to classify ILs in the different response groups (from A to D) according to the concept developed by Fernandez (1992). Contrasting groups A (high resilient/tolerant and high productive ILs) and D (low resilient/tolerant and low productive ILs) were compared for image-based and manually measured traits.

### Data Analysis

The data from phenotyping platform are used to determine the overall importance of the factors like genotype, treatment and their interaction for the observed digital and manual traits, follows a Gaussian linear mixed model which formulated for each trait separately: *y* = *Xβ* + *Zu* + 𝜀, where *y* is the response variable with *n* observations of a given continuous trait, *X* and *Z* are design matrices of fixed and random effects, respectively; *β* and *u* are the estimated and predicted parameters of the fixed and random effects in the model; 𝜀 is the vector of the random errors associated with the response variable. The effects of the replication in the model was treated as fixed while the effects of genotype, treatment and the interaction as random effects. For the random terms in the model the normal distribution is assumed with *E*(*u*) and *E*(𝜀) equal to zero and variance-covariance matrices *G* and *R* side of the model. In order to ensure the reliability of the models, the AOM algorithm based on the Studentized residuals for the detection of the anomaly or extreme observations along with diagnostic plots were used. The significance of the fixed effect model term was assessed using Wald test, whereas the significance of the random term by likelihood ratio (LR) test. In order to account for different precision of the treatment conditions, we fitted two competitive models for each response variable: (i) with homogeneous residual error variances across the treatments and (ii) with heterogeneous residual error variance across the treatments. The selection among the competitive models was made according to Akaike Information Criterion (AIC). The model with the lower AIC value was selected. In addition, the random effects (i.e., the BLUPs) of the genotypes were predicted in each treatment and used for all subsequent analyses.

The measures of descriptive statistics, box-plot as well as the Person linear correlation coefficients among the observed traits were used. Furthermore, the correlation network map based on the matrix of Person linear coefficients was constructed to visually identify the correlation pattern that is not observable in a symetric correlation matrix (Ursem et al., 2008). In a correlation network map, the traits represent variables as nodes which are connected by edges, whose width is proportional to the strength of the correlation. Based on the REML estimates of the variance components of random terms, the sample-basis heritability (*h*^2^) of the traits is estimated using the following equation (Holland et al., 2003): h^2^ = ${\hat{\sigma }}_{G}^{2}$/${\hat{\sigma }}_{P}^{2}$, where is ${\hat{\sigma }}_{G}^{2}$ – estimated genetic variance; ${\hat{\sigma }}_{P}^{2}$ – total phenotypic variance expressed as the sum of ${\hat{\sigma }}_{G}^{2}$ – genetic variance component, ${\hat{\sigma }}_{\mathrm{GT}}^{2}$ – genotype × treatment variance component and a ${\hat{\sigma }}_{\varepsilon }^{2}$ error variance.

In order to interpret the importance of the traits on biomass in two treatments, the multivariate regression approaches were used. Due to previously observed high correlations among the traits which are considered as the predictors (*j* = 1, …, *k*) of the biomass variation, we fitted two alternative shrinkage/penalized regression approaches known as the ridge regression (RR) model (Hoerl and Kennard, 1970) and the Least Absolute Shrinkage and Selection Operator (LASSO) regression model (Tibshirani, 1996). In the RR model, the minimization of the residual sum of squares is based on the following equation:

β^RR=arg min β RRS(β)=arg min β{∑i=1n(yi− β0−∑j=1kxij βj)2+ λ∑j=1k βj2}

where λ ≥ 0 is the complexity parameter which controls the amount of the shrinkage, and $\ell_{2}=\sum_{j=1}^{k}\beta_{j}^{2}$ is the ridge penalty function (Hastie et al., 2009).

The LASSO model uses a different type of penalty function and minimizes the residual sum of squares based on the following equation:

β^LASSO=arg min β{∑i=1n(yi− β0−∑j=1kxij βj)2+ λ∑j=1k| βj|}

where $\ell_{1}=\sum_{j=1}^{p}\left|\beta_{j}\right|$ is the LASSO model penalty function (Hastie et al., 2009). In contrast to RR model penalty function, the LASSO model penalty function enable an efficient shrinking of some of the regression coefficients (i.e., ${\hat{\beta }}_{\mathrm{LASSO}}$) to zero. Thus, the LASSO model usually results into sparse models that are easier to interpret. For RR and LASSO models, the optimal value of the complexity parameter was estimated by fivefold cross-validation.

Diagrams were used to group the ILs in terms of RCI and PCI into four groups (from A to D). The diagram axes were generated by the means of all ILs and the values of each IL distributed into quadrants. Means between contrasting groups A and D were separated by *t*-test. The two-way table of genotype-by-treatment predictions for biomass data was analyzed by the interaction AMMI model (Cornelius et al., 2001). The table was double-centered such that each genotype (*g*_i_) biomass in each treatment (*t*_j_) value has an interaction value (*tg*_ij_), i.e., y_ij_ -ȳ_i._ -ȳ_.j_ + ȳ_.._ where *y_ij_* – is the effect of *i*-th genotype in *j*-th treatment; ȳ_i._ – effect of *i*-th genotype; ȳ_.j_ – effect of *j*-th treatment; ȳ_.._ – overall mean. The singular value decomposition method was used to derive the hypothetical parameters of the AMMI model. The derived hypothetical parameters are displayed on two-dimensional biplot graph (Bradu and Gabriel, 1978).

All computations and data visualizations were accomplished within the R computing environment (R Core Team, 2019). The mixed model analyses were conducted with the ASReml software (Gilmour et al., 2009).

## Results

### Trait Performance and Estimation of Variance Components

For all architectural (except Rnd) and biomass-related traits means were the highest in C, followed by N, then by W and N + W (Supplementary Table 4). The same stands for fluorescence-based traits (FI and PSII). Average reduction of architectural, biomass-related and flourescence-based traits due to nitrogen stress (N), water stress (W), and combination of nitrogen and water (N + W) stress was 3.2% (ranged 0–7%), 19.3% (ranged 1–44%), and 21.8% (ranged 1–46%) (Supplementary Table 4). In all stress treatments the highest percentage reduction was found for BDw (ranged 7–46%), followed by EBv (ranged 7–42%), and BFw (ranged 6–38%). The lowest percentage reduction associated with applied stresses was found for Rnd (ranged 0–1%) and Sol (ranged 0–4%). For color-related traits (except RGB_g) means in N + W and W treatments were higher compared to control conditions (C), while differences between N and C were mostly marginal. Color ratios (Y2G, B2G, and R2G) of stressed plants under W and N + W had highest change of means among all image-based and manually measured traits compared to control plants (ranged from 0.5- to 4.5-fold increase).

Boxplots illustrating the phenotypic distributions within treatments for each image-based and manually measured trait are provided as Supplementary Figures 2, 3, respectively. Considering coefficient of variation (CV), most of the studied traits showed relatively low (5–10%) and moderate (10–20%) variability within treatments (Supplementary Table 5). High variability (at least 25% in any of the treatments) was determined for B2G and TCom, with R2G being by far the most variable trait (varying from 55 to 83% for different treatments). Very low variability (<5%) in each of the treatments was exhibited by color-related traits Lab_a, Lab_ b and RGB_g as well as RWC. For most of the traits CV was similar among the treatments. However, there were several exceptions. Variability of PSII, color ratios and RWC were increased in conditions with water stress (W and N + W) compared to conditions where water stress was not applied (C and N). On the other side, variability of biomass-related traits BFw, BDw and SPW was gradually declined with increasing the level of stress.

**FIGURE 1:**
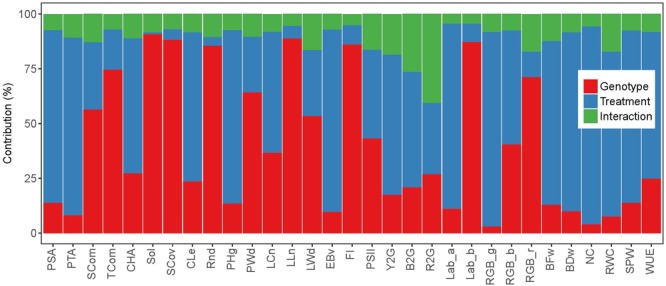
Relative contribution of the variance components (estimated by REML model) to the phenotypic variance of image-based and manually measured traits.

Highly significant (*P* < 0.001) REML variance components of treatment, genotype and interaction effects were obtained for almost all image-based and manually measured traits (Supplementary Table 6). The only exceptions were Sol in a case of treatment effect (*P* > 0.05) and RWC in a case of genotype effect (*P* < 0.01). Studied traits showed variable genotypic and treatment effects and their interactions (Figure 1 and Supplementary Table 6), with dominant effect of treatment for 12 image based traits such as RGB_g (89%), Lab_a (85%), EBv (83%), PTA (81%), etc. Genotype was accounted for most of the variation for twelve image-based traits, being highest for Sol (91%), LLn (89%), SCov (88%), Lab_b (87%), FI (86%), etc. The only trait with dominant interaction effect was R2G (41%). Variation of all manually measured traits was dominated by treatment effect. Heritability (*h^2^*) was generally slightly higher for image-based traits than for manually measured traits (Supplementary Table 6). Highest heritabilities (over 0.96) were found for some architectural traits such as TCom, Sol, SCov, and LLn, as well as FI and Lab_b. Lowest heritabilities were obtained for some physiological traits such as RGB_g (0.56), RWC (0.63), NC (0.70), and R2G (0.73), as well as PTA (0.74).

### Major Correlations Between Image-Based and Manually Mesured Traits

**FIGURE 2:**
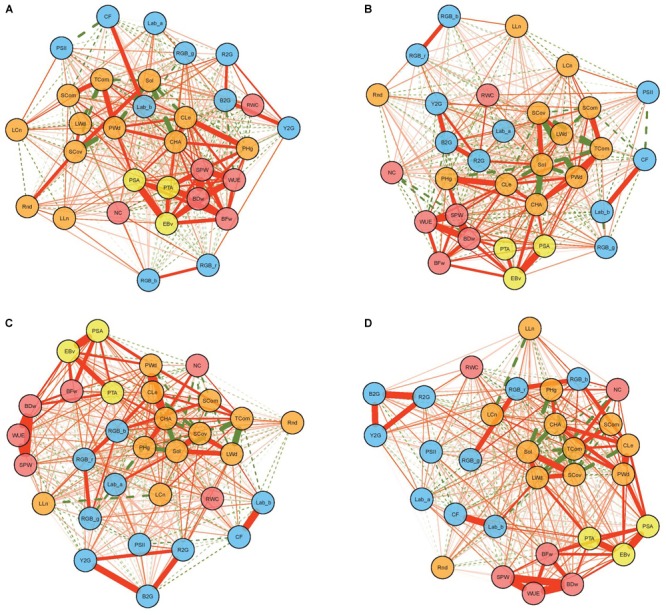
Phenotypic correlation networks among 25 image-based and six manually measured traits in control **(A)**, nitrogen stress **(B)**, water stress **(C)**, and combination of nitrogen and water stress **(D)** treatment. Full and dotted lines represent positive and negative correlations, respectively. Line width is proportional to the strength of the correlation. Red, yellow, orange, and blue nodes represent manually measured traits, biomass-related traits, architectural traits, and physiological traits, respectively.

Networks visualizing phenotypic (*r*) correlations among all studied traits in four treatments are given in Figure 2. Several manually measured traits showed distinct relationships with image-derived traits regarding different treatmans. For example, NC had significant correlations with image-derived traits only in treatments with water stress, while the oposite was true for RWC and SPW. In general, higher number of significant correlations between image-derived and manually mesured traits were found in non-water stress treatments (C and N) than in treatments with water stress (W and N + W) (Supplementary Tables 7–10). Significant positive correlations were observed between two types of biomass measurements (BFw and BDw) and image-derived estimated biovolume (EBv) in all treatments, with the highest in C (*r* = 0.753 and 0.793, respectively), followed by N (*r* = 0.632 and 0.635, respectively), then W (*r* = 0.613 and 0.545, respectively) and N + W (*r* = 0.615 and 0.537, respectively). Further image-derived biomass-related traits, PSA and PTA, respectively, showed relationships with BFw and BDw in a similar patterns like EBv. BDw had also strong positive relationships with CHA, Cle, and PHg, but only in C and N treatments. Apart from BFw and BDw, EBv had significant positive correlation with WUE (in all treatments) and SPW (only in C and N).

### Variable Selection

To define a subset of image-derived traits that contribute to BFw and BDw as dependent variables in the model, LASSO and Ridge regressions were applied. These techniques were chosen to avoid the problem of the high degree of the multicollinearity as indicated by high correlation coefficients (*r*) among predictors (image-derived traits) (see Figure 2 and Supplementary Tables 7–10). In this study, we focused on the results from the LASSO model selection approach as results from the two different methods were well consistent and led to similar conclusions. Results from the Ridge regression are provided as supplementary material (Supplementary Table 11). The LASSO estimates of the contributors to BFw and BDw in each treatment among 25 image-based traits are presented in Table 2. The number of non-zero coefficients for BFw was highest in C and N (both 12) and the lowest in W and N + W (both nine). Contrary to this, for BDw the highest (13) and the lowest (8) number of non-zero coefficients were obtained for W + N and C, respectively. In general, biomass-related traits such as EBv and PTA showed to be among the most significant contributors to BFw and BDw in each treatment. Further traits that were contributors in each treatment were color ratios (Y2G and B2G) and PSA for BFw, and SCom, B2G and Lab_b for BDw. However, several traits showed distinct patterns of trait importance between tretments. Namely PSII, Lab_a, Sol, and RGB_b had non-zero coefficients for BDw in water stress treatments (W and N + W), but not under mild (N) or non-stress (C) conditions. The same is true for PSII, Lab_a and Rnd in a case of BFw. On the other hand, Cle and SCov were significant contributors for BFw and BDw, respectively, only in control treatment. Several image-based traits such as CHA, PWd, FI, and RGB_g were not substantially important for BFw and BDw in any treatment.

**Table 2:** Estimated coefficients from the LASSO applied to the biomass fresh and dry weight.

Predictor	Biomass fresh weight (BFw)				Biomass dry weight (BDw)			
	C	N	W	N + W	C	N	W	N + W
PSA	0.049	0.084	0.301	0.232			0.087	0.046
PTA	0.424	0.532	0.237	0.184	0.269	0.228	0.395	0.294
SCom					–0.146	–0.164	–0.126	–0.151
TCom	–0.162	–0.633						
CHA								
Sol							0.143	0.102
SCov					0.073			
CLe	0.033					0.182		0.041
Rnd			–0.221	–0.146	0.096	0.077		
PHg	0.171	0.344			0.304	0.203		
PWd								
LCn		–0.011	–0.047	–0.023		–0.035	–0.196	–0.111
LLn						0.024	0.090	0.124
LWd	–0.210	–0.435						
EBv	0.340	0.218	0.212	0.260	0.337	0.236	0.225	0.246
FI								
PSII			0.346	0.316			0.194	0.176
Y2G	0.124	0.153	–0.177	–0.151				
B2G	–0.048	–0.060	–0.051	–0.033	–0.033	–0.102	–0.090	–0.094
R2G	0.062	–0.008						
Lab_a			0.089	0.039			0.114	0.082
Lab_b	–0.098	–0.332			–0.174	–0.224	–0.192	–0.182
RGB_g								
RGB_b	–0.061	–0.037					0.026	0.075
RGB_r	0.031	0.005						

*The blank entries correspond to variables excluded from the model. C, control; N, nitrogen stress; W, water stress; N + W, combination of nitrogen and water stress. Trait names are given in Section “Materials and Methods,” while their definitions and details of extraction are provided in Supplementary Table 2.*

### Identification of Stress-Adapted Genotypes Within the Investigated Population

**FIGURE 3:**
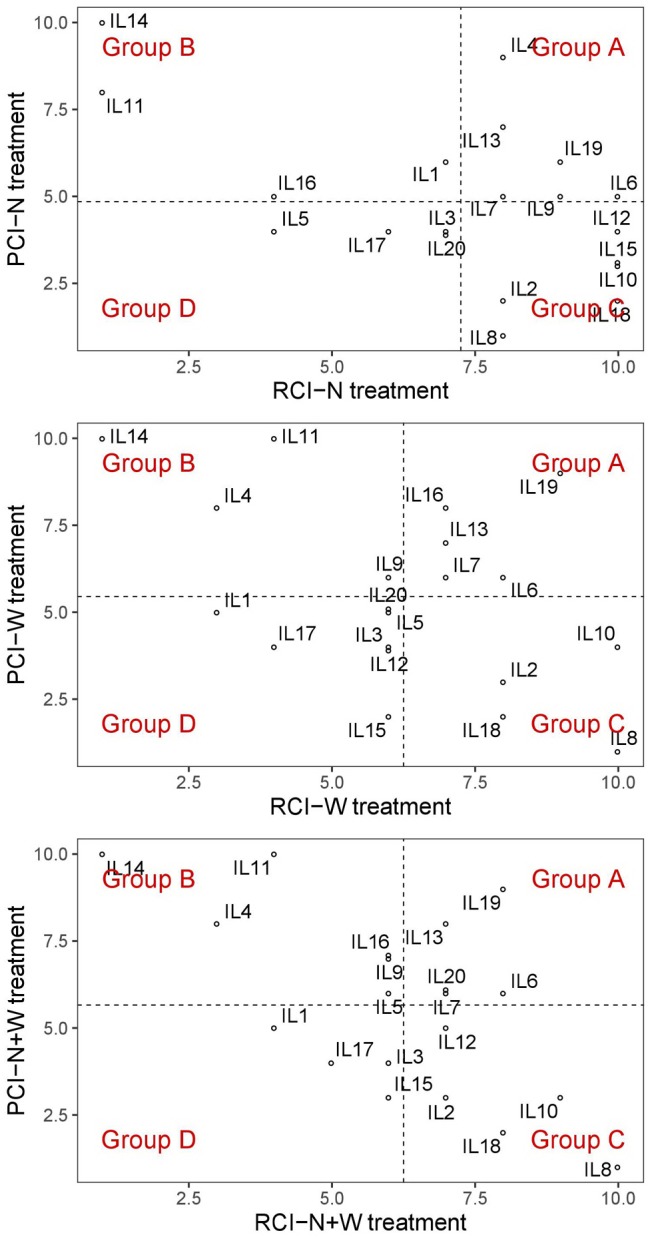
Distribution diagram of twenty maize inbred lines (ILs) into four different response group (from A to D) according to their variation in resilience capacity index (RCI) and productive capacity index (PCI) calculated for nitrogen stress (N), water stress (W), and combination of nitrogen and water stress (N+W) treatments.

As a first step toward selection of stress-adapted genotypes, we used five stress-tolerance indices (SSI, TOL, MP, GMP, and STI) based on dry biomass weight to summarize the genotypic response to the applied stresses (Supplementary Tables 12–14). In the next step, for each of the five indices a simple 10-grading (scoring) assesment of ILs was applied to obtain RCI and PCI (Supplementary Tables 15–16). Distribution of ILs in terms of variation in RCI and PCI was used to construct a diagram, showing the separation of the ILs into four groups from A to D (Figure 3 and Supplementary Table 17) of differential response under stress conditions according to concept of Fernandez (1992). Groups A (best genotypes-high tolerant and high productive) and D (worst genotypes- low tolerant and low productive) represent the extremes, which are in the focus of this study. We looked for statistical difference between average values of digital and manually measured traits for groups A and D in each stress treatment and control (Table 3).

**Table 3:** Comparison of best (A) and worst (D) inbred lines based on their productivity and tolerance under stress (A, high productivity and high tolerance; D, low productivity and low tolerance).

Trait code	N treatment		W treatment		N + W treatment	
	A (*n* = 5)	D (*n* = 4)	A (*n* = 5)	D (*n* = 4)	A (*n* = 5)	D (*n* = 4)
**Image-based traits**					
PSA	1312 ± 30	1295 ± 22	**1186 ± 29^∗^**	**1052 ± 39**	1091 ± 36	1004 ± 34
PTA	1546 ± 25	1449 ± 61	**1191 ± 30^∗∗^**	**1006 ± 40**	**1107 ± 41^∗∗^**	**893 ± 21**
SCom	925 ± 27	962 ± 66	857 ± 39	910 ± 72	796 ± 17	813 ± 76
TCom	509 ± 44	546 ± 80	445 ± 56	472 ± 68	404 ± 32	442 ± 91
CHA	5512 ± 276	5321 ± 328	4226 ± 258	3984 ± 250	3823 ± 155	3786 ± 242
Sol^†^	28.5 ± 1.5	27.7 ± 2.4	28.4 ± 1.5	2.56 ± 1.1	**28.7 ± 1.2^∗^**	**23.5 ± 1.3**
SCov^†^	13.8 ± 0.8	13.4 ± 2.1	**13.7 ± 0.4^∗^**	**11.5 ± 0.9**	12.6 ± 0.5	11.3 ± 0.1
CLe	**83.3 ± 2.4^∗^**	**74.1 ± 3.2**	68.0 ± 1.8	65.3 ± 2.3	66.6 ± 1.8	64.4 ± 2.8
Rnd	0.75 ± 0.02	0.74 ± 0.02	0.72 ± 0.03	0.72 ± 0.01	0.69 ± 0.02	0.71 ± 0.01
PHg	143 ± 6	137 ± 7	98 ± 5	101 ± 4	96 ± 6	99 ± 6
PWd	65.0 ± 1	62.8 ± 4	63.3 ± 2	59.1 ± 3	61.5 ± 2	58.7 ± 4
LCn	11.8 ± 0.3	12.8 ± 0.7	10.2 ± 0.5	10.9 ± 0.7	9.6 ± 0.7	10.1 ± 1.1
LLn	69.1 ± 3.4	66.6 ± 4.1	68.8 ± 3.5	65.1 ± 4.1	68.7 ± 4.5	66.4 ± 2.7
LWd	7.7 ± 0.1	6.9 ± 0.4	6.8 ± 0.2	6.5 ± 0.3	6.8 ± 0.2	6.2 ± 0.4
EBv^††^	**154 ± 4^∗^**	**139 ± 1**	**115 ± 5^∗^**	**94 ± 4**	**102 ± 3^∗^**	**86 ± 2**
FI^†^	31.2 ± 0.8	30.4 ± 1.1	30.4 ± 0.7	28.4 ± 1.5	30.2 ± 0.7	27.0 ± 2.1
PSII^†^	52.1 ± 0.5	51.5 ± 0.7	46.8 ± 0.7	46.1 ± 1.7	**46.6 ± 0.6**	**43.9 ± 2.7**
Y2G^†^	7.4 ± 0.2	8.0 ± 0.2	9.8 ± 0.6	10.4 ± 1.0	10.1 ± 0.7	10.9 ± 1.7
B2G^†^	1.9 ± 0.1	1.8 ± 0.1	3.2 ± 0.3	3.4 ± 0.7	3.4 ± 0.3	3.5 ± 1.1
R2G^†^	2.0 ± 0.0	2.0 ± 0.0	4.0 ± 0.1	5.0 ± 0.2	6.0 ± 0.1	6.0 ± 0.3
Lab_a	106 ± 1	106 ± 1	111 ± 1	111 ± 1	111 ± 1	110 ± 1
Lab_b	152 ± 1	155 ± 1	154 ± 1	155 ± 1	155 ± 1	155 ± 1
RGB_g^†^	43.8 ± 0.6	41.6 ± 0.6	34.6 ± 0.6	34.3 ± 0.7	34.2 ± 0.9	34.7 ± 0.9
RGB_b^†^	16.6 ± 0.2	16.2 ± 0.3	18.0 ± 0.5	17.6 ± 0.6	17.8 ± 0.6	17.4 ± 0.4
RGB_r^†^	26.3 ± 0.5	25.8 ± 0.4	27.7 ± 0.6	26.7 ± 0.6	27.3 ± 0.8	26.1 ± 0.2
**Manually measured traits**					
BFw	215 ± 2	200 ± 8	**156 ± 2^∗^**	**136 ± 6**	**146 ± 2^∗^**	**124 ± 6**
BDw	**24.4 ± 0.8^∗^**	**21.3 ± 0.3**	**15.4 ± 0.4^∗∗^**	**12.9 ± 0.3**	**14.4 ± 0.4^∗∗^**	**12.1 ± 0.1**
NC	1.78 ± 0.07	1.79 ± 0.05	2.73 ± 0.08	2.79 ± 0.10	2.63 ± 0.12	2.61 ± 0.13
RWC	93.8 ± 0.5	93.2 ± 0.5	85.8 ± 1.2	83.9 ± 1.1	**87.2 ± 1.1**	**80.9 ± 1.5**
SPW	**77.2 ± 1.7^∗^**	**68.4 ± 1.2**	**50.1 ± 1.0^∗^**	**45.5 ± 1.1**	**50.6 ± 2.2^∗^**	**43.8 ± 1.0**
WUE	1.54 ± 0.03	1.49 ± 0.03	**2.11 ± 0.06^∗^**	**1.84 ± 0.04**	**2.04 ± 0.05^∗^**	**1.75 ± 0.05**
**Indice**					
PNUE	**12.3 ± 1.7^∗∗^**	**24.1 ± 1.0**	93.0 ± 10.0	102.1 ± 5.0	72.0 ± 8.0	77.1 ± 2.0

*n denotes number of inbred lines belong to the group. †Values were multiplied by 100, ††given in mega-voxel. Significant differences between groups A and D obtained by *t*-test were bolded (^∗^significant at *P* < 0.05; ^∗∗^significant at *P* < 0.01). N, nitrogen stress; W, water stress; N + W, combination of nitrogen and water stress. Trait names are given in Section “Materials and Methods,” while their definitions and details of extraction are provided in Supplementary Table 2.*

**FIGURE 4:**
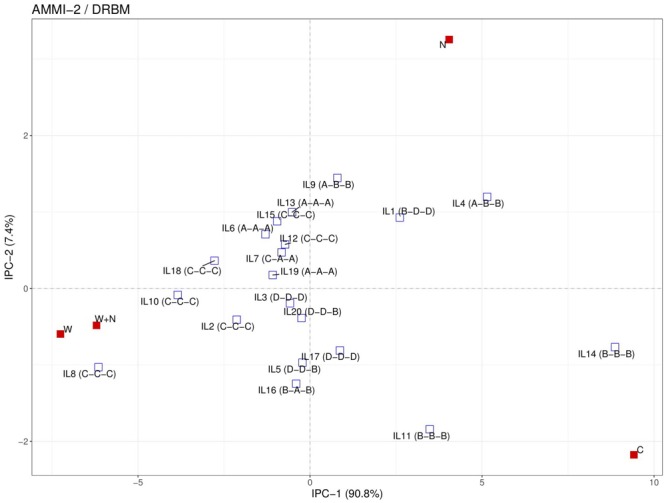
Additive main effects and multiplicative interaction (AMMI) 2 biplot for 20 inbred lines (IL) based on measured dry weight in four treatments (C = control; N = nitrogen stress; W = water stress; N + W = combined nitrogen and water stress). Letters in brackets signify belonging of ILs to the different response groups (from A to D) according to their variation in resilience capacity index and productive capacity index calculated for N, W, and N + W treatment, respectively. Details of the inbred lines are provided in Table 1.

In the N treatment, the five ILs of group A (IL4, IL6, IL9, IL13, and IL19) had significantly higher EBv, BFw, BDw, PNUE and SPW than the four ILs of group D (IL3, IL5, IL17, and IL20). In the W treatment, the five ILs group A (IL6, IL7, IL13, IL16, and Il19) showed significantly higher PSA, PTA, SCov, EBv, DFw, BDw, SPW, and WUE than the five ILs of group D (IL1, IL3, IL5, IL17, IL20). In the N + W treatment, group A consisted of four ILs (IL6, IL7, IL13, and IL19) that had significantly higher PTA, Sol, EBv, DFw, BDw, SPW, WUE, and RWC than the three ILs of group D (IL1, IL3, and IL17). The differences expressed under the stress treatments between groups A and D were not observed in control treatment for any trait. Genotype × treatment interaction analysis for dry weight showed to be highly consistent with the classification of the ILs into the four groups (Figure 4). ILs placed in the left upper quadrant of the biplot can be regarded as stable since their vectors had no acute angles with vectors of any treatment (e.g., ILs 6, 7, 12, 15, etc.). These ILs were grouped in all treatments either in group A (marked as A–A–A on the biplot) or C (marked as C–C–C on the biplot). Three ILs placed in upper right quadrant (9, 1, and 4) appeared to be most adapted to mild stress (N). Most adapted to severe stress (W and N + W) conditions are ILs 10, 8, and 2. They were classified in group C in all treatments (C–C–C). In general, in the biplot the 20 ILs showed no grouping according to their origin, maturity group or gene pool. This was also not the case for five ILs (9, 11, 12, 13, and 14) identified to carry unique SNP mutations in *MYBE1* transcription factor gene involved in drought stress tolerance pathways.

## Discussion

Expression of maize growth- and stress tolerance-related traits was monitored during the vegetative period from approx. the 4-leaf stage up to the 14-leaf stage by four types of imaging modules, visible (color), near-infrared, fluorescence (static bulk), and kinetic chlorophyll fluorescence (at one day). This multi-sensor setup supports the assessment of approx. 200 phenotypic traits and thus offers novel opportunities to gain knowledge of genetic determinants and mechanisms governing plant performance under challenging environmental conditions. However, it also imposes a substantial challenge with respect to the analysis of the large volume of data. In this study, we focused on 25 image-derived traits which are highly informative (showed genotype and treatment effects) and with biological meaning (can be used as a proxy for important agronomic features). The majority of the extracted traits were derived from RGB images (except two FLUO-based traits), which is by far the most frequently used imaging modality in phenotyping experiments (Ge et al., 2016). Color imaging can be used not only to assess growth status and biomass accumulation of plants but also their nutritional or health status, while fluorescence imaging detects chlorophyll and other fluorophores signals and can serve as a proxy for stress symptoms (Chen et al., 2014; Janka et al., 2018). Selected traits are broadly classified as of architectural, biomass-related and physiological type. For architectural and biomass related traits we focused on the values of the final measurement at the end of the experiment to correlate them with manually measured traits, including destructively determined fresh and dry biomass weight. However, for the traits that represent stress symptoms (i.e., color-related traits), data from the day of the maximum water stress were used instead, as these traits tend to change over time (Neumann et al., 2015).

While the applied water stress level built up rapidly and was quite severe, the applied nitrogen stress appeared to be in a mild and more chronic fashion (our intention was to obtain moderately reduced nitrogen growth conditions). In other words, under the low N treatment the plants ran more slowly into N-deficiency and had probably more time to adjust than in the case of water stress. This resulted in less observable phenotypic effect in the N treatment compared to W and N + W treatments. As expected, biomass-related traits were in general the most sensitive to applied stresses among studied image-derived traits, except for color ratios (Y2G, B2G, and R2G). Variance component analyses revealed that heritability of image-derived traits was high for most of them, with values of 0.56 and above, especially for architectural traits. Chen et al. (2014) also reported higher heritability of geometric and morphological than physiological traits obtained from digital images in barley. Biomass yield is a quantitatively inherited trait and its heritability tends to be low due to interactions with several other traits and a high environmental influence (Jackson et al., 1996). However, in this study the heritability of estimated biovolume, the proxy of biomass, was estimated to be rather high (80–84%), due to low interaction (genotype × treatment) effect. This is consistent with previous findings by Junker et al. (2015) and Muraya et al. (2017) in the same facilities, who found non-significant genotype × cultivation interaction for biomass in both smaller and larger panel of maize lines, respectively. High heritability of image-derived traits is promising to study the genetic architecture of maize plant growth (Muraya et al., 2017; Zhang et al., 2017).

### The Pattern of Phenotypic Trait Correlations

The structure of phenotypic correlations including six manually measured was assessed at the end of the experiment. Consistent with previous studies in different crop species, the digital image-derived traits proved to be reliable estimators of manually measured traits (Humplík et al., 2015; Neilson et al., 2015). The correlation coefficients between estimated biovolume (biomass proxy) and measured fresh and dry weight obtained in this study were highly significant but a little lower, particularly under stress (ranged from *r* = 0.62 and 0.61 in combination of nitrogen and water stress to *r* = 0.75 and 0.79 in control treatment, respectively), compared to previously reported data in barley and maize (Chen et al., 2014; Honsdorf et al., 2014; Ge et al., 2016; Muraya et al., 2017). The correlation coefficient depends on the range of trait values displayed by the lines under analysis. In contrast to Muraya et al. (2017), the lines investigated in the present study have been pre-selected to enrich the elite MRIZP breeding material. The different composition of the panels and the assessment of the traits at a later developmental stage (at 50 days vs. 41 days of cultivation) well explains the lower estimates in our study, as the correlations become weaker at later growth stages when the difference in plant architecture of genotypes becomes more pronounced (Ge et al., 2016). Gradual decrease of correlation coefficients between image-derived and manually measured biomass with increasing stress intensity suggests that different physiological mechanisms and genes are involved in adaptation for higher stress (Banziger and Edmeades, 1997).

Phenotypic correlations within traits of the same category (i.e., architectural and biomass-related traits) were mainly high and positive, while physiological traits were either not correlated or negatively correlated with other traits. This is in accordance with Chen et al. (2014) who suggested that the variation in color-based traits has an independent genetic basis from other traits. As expected, estimated biovolume had the highest correlation coefficients with fresh and dry weight among image-derived traits in all treatments, followed by two other biomass-related traits, projected plant area from top and side view. Furthermore, these were the only image-derived traits significantly related with fresh and dry weight in W and N + W treatments.

### Subset of Image-Derived Traits That Relate to Fresh and Dry Biomass

To identify the most important image-derived traits related to mid-season biomass accumulation for breeding purpose we applied two regression techniques that have been extensively used for model selections and feature reductions in machine learning literature and applications (Aloraini, 2017). As many predictors might have weak predictive value relative to the noise in the data, shrinkage would be appropriate for the stabilization of the estimates (Kruschke, 2015). In this study, the focus is on the results from the Lasso regression that performs feature selection along with shrinking coefficients, although data from the Ridge analysis, that keeps all variables in the model and shrinks the coefficients toward zero, was rather consistent.

In general, the number of identified non-zero coefficients by Lasso model for fresh and dry weight versus image-derived traits was higher than the number of significant simple correlations coefficients between the same group of traits, especially under more stressed treatments (W and N + W). Again, biomass-related traits (EBv, PSA, and PTA) were among the most important traits both for biomass measurements in all treatments. Under severe stress conditions (W and N + W) Lasso analysis identified several additional important physiological and architectural traits.

In water stress treatments photosystem II operating efficiency (PSII, a proxy for photosynthetic efficiency) was most important among all image-derived traits to distinguish genotypes with high/low fresh weight. For dry weight, PSII is the most important after EBv, PSA, and PTA. PSII was also among the top ranked traits for fresh and dry weight under severe stress (W and N + W) identified by Ridge analysis. PSII is based on pulse-amplitude modulated technique which allows early analysis of activity and regulation of photosystem II, even before visible symptoms of biotic and abiotic stresses become apparent (Humplík et al., 2015; Tschiersch et al., 2017). In *Arabidopsis*, severe drought stress has been shown to reduce PSII efficiency (Jansen et al., 2009). In contrast to PSII, the static bulk fluorescence parameter measured in this study (FI) was neither important for fresh nor for dry weight in any treatment. It may distinguish non-stressed and senescent leaves at later stages of stress progression as reported by Humplík et al. (2015). Along with the aforementioned traits also several architectural and color-related traits were involved in the Lasso models for fresh and dry weight in stress treatments. While architectural traits refer to shape, length or area of the whole plant or part of the plant, color traits may be related to physiological responses and to the degree of tissue damage. In severe stress treatments (W and N + W), the most important color-related trait for fresh weight was yellow to green (Y2G) color ratio, which may indicate the degree of wilting symptoms. This trait was reported to be the most sensitive to drought among several color traits in a study of vegetative biomass accumulation in barley (Neumann et al., 2015). Interestingly, Y2G appeared to be of some importance also in C or N conditions. In contrast to severe stress conditions, here, Y2G ratio was positively correlated with fresh weight. It might be related to a phenomenon called physiological leaf spotting or flecking, which is the mild, genetically determined spotting (lesion) commonly observed on the leaves of maize (Vontimitta et al., 2015; Olukolu et al., 2016) including line Mo17 (Zehr et al., 1994) and in several other cereals (Nair and Tomar, 2001; Behn et al., 2004). Moderate coefficient of variation for Y2G ratio in the C treatment (over 11%) suggests genetic variation of this trait independent of stress.

The most important color-related trait for dry weight in all treatments was Lab_b. Despite low variation in all treatments (2.4–2.7%), significant differences of this trait were observed among the studied genotypes. Genotypes with high values of this trait (which indicate yellow color) tend to have low dry weight. Furthermore, Lab_b was top-5 ranked for dry weight by Ridge regression in all treatments, even higher than EBv. Lab_b was also among most important and top-ranked image-based traits for fresh weight, particularly in C and N treatments (in which Y2G was not so prominent). Thus, average color in the b^∗^ range (blue to yellow) of the L^∗^a^∗^b^∗^ color space (Ibraheem et al., 2012) can be regarded as overall the most useful among studied color-related traits to screen genotypes for vegetative biomass accumulation under different conditions.

While several traits (e.g., PTA, EBv, and B2G) were informative for fresh *and* dry weight in all treatments, others showed large differences in their importance for the two measures of biomass: The aforementioned Y2G may be important for predicting fresh weight but not for dry weight, whereas side compactness (SCom) showed the reverse. In general, decreased plant height and plant width, thus higher SCom, was in negative correlation with dry weight in all treatments.

Other architectural traits detected by both analyses as being important for dry weight only in stress treatments, were all related to leaf traits (LCn and LLn): Maize plants with few (LCn) but long leaves (LLn) tended to have high dry weight, which is in agreement with the recent indication of leaf length as one of the key aspects (along with leaf angle, curvature and dark green color) for ideotype-based maize breeding (Zhang et al., 2017). The relation of leaf number with biomass observed in stress treatments but not in C conditions may be linked to variation in developmental progression among the investigated genotypes: While at the time of transplanting the maximum difference in leaf number genotypes was only 0.8, it was almost four leaves at the end of the experiment in each treatment. Thus, more rapidly developing genotypes were exposed to the stress during more advanced stages. Actually, few of the genotypes were at reproductive stage (tasseling) at the end of the experiment, which is often considered more sensitive to abiotic stress than vegetative stage (Çakir, 2004; Walsh et al., 2012) and flowering time as a key to local environmental adaptation (Li et al., 2016). Solidity (Sol) is another leaf trait found to be important for identifying genotypes with high dry weight accumulation under severe stress conditions (W and N + W), but not in mild stress (N) or non-stress conditions (C). This trait measures the degree of leaf area coverage and can be used as a proxy trait of the agronomic measure of LAI (Neilson et al., 2015). LAI (leaf area per unit growth area) is a key determinant of radiation interception, biomass accumulation and yield in maize (Lindquist et al., 2005; Lukeba et al., 2013). In contrast to Sol, PHg was important for measured biomasses only under C and N, but not under severe stress (W and N + W). These findings are in accordance with Chen et al. (2018) who suggested that image-based phenotypic traits reflect differences in underlying determinants of plant biomass subjected to various growing conditions.

### Comparing Contrasting Inbred Lines for Image-Derived and Manually Measured Traits

To further investigate which image-derived trait might be of interest for maize breeders and researchers regarding mid-season stress adaptation, we first classified the genotypes in terms of yield formation (here dry biomass production) under stress and then searched for image-derived and manually measured traits with significant differences among the groups. Classification into four different response groups (from A to D) was done for each treatment according to Thiry et al. (2016) by analyzing the RCI, in terms of dry biomass decrease of ILs under stress within a population, compared to non-stress conditions, and the PCI, in terms of mean production of ILs under both stress and non-stress conditions within a population. With the assumption that contrasting groups A (best ILs with a high value in both indices) and D (worst ILs with a low value in both indices) would differ in traits underlying stress adaptation, they were interrogated for traits with significant differences in expression.

In the N treatment, groups A and D significantly differed for two image-based traits (EBv and CLe) and three manually measured traits (BDw, SPW, and PNUE), in all cases in favor of the best ILs. Since indices RCI and PCI were based on dry weight, the difference between groups A and D in biomass-related traits (EBv, SPW, and BDw) was expected. CLe describes the maximum diameter of the plant which is very informative (Honsdorf et al., 2014) as plants with a large diameter cover a larger area, tend to be bigger, have a higher growth rate and a higher biomass than plants with a smaller diameter. Also both regression analyses indicated this trait as important for dry weight in the N treatment. The detected image-derived traits will be suitable for high-throughput measurement of varietal differences in dry matter accumulation and nitrogen use efficiency at the vegetative stage. This could be advantageous as it is unaffected from additional variables affecting (seed) yield in later stages such as number of grain per ear and mass of 1000 grains (Namai et al., 2009) and can help in speeding up the phenotyping process for testing hybrids as well as inbreds (Ciampitti et al., 2012).

In the water stress treatments (W and N + W), all biomass-related traits (except PSA in N + W treatment) significantly differed between groups A and D and WUE was significantly higher in the A vs. the D group. Along with drought tolerance, WUE is one of the two primary mechanisms of adaptation to water deficit (Condon et al., 2004). The biomass-related image-derived values are among the strongest criteria for identifying plants with high resilience and high productivity under severe stress. In addition, the architectural trait surface coverage (SCov) was also significantly higher in the best ILs compared to the worst ones in the W treatment. SCov can be used as a proxy for LAI, particularly when plants were subjected to water-limiting conditions (Neilson et al., 2015). Contrary to SCov, two previously discussed image-based traits Sol and PSII appeared to make substantial difference between the two contrasting groups in N + W treatment, but not in W treatment. Also, RWC is significantly different between A and D group only in combination of nitrogen and water stress, but not in water stress solely. These differences between W and N + W stresses are in accordance with the well-known fact that the response of plants to a certain combination of stresses is unique and cannot be directly extrapolated from the response of plants to individually applied stresses (Mittler, 2006; Humbert et al., 2013).

Interestingly, when contrasting groups from particular stress treatments were compared in control conditions, only Y2G of all studied traits appeared to be significantly different between groups. In stress-free conditions, the best ILs from each stress treatment had significantly lower Y2G than the worst ILs. As mentioned before, Y2G might be affected by naturally occurring mild leaf spotting, which is possibly related to disease resistance (Vontimitta et al., 2015; Olukolu et al., 2016), but which could also lead to reduced growth and to a yield penalty (Todesco et al., 2010; Olukolu et al., 2016).

### Adaptation and Biomass Yield Stability of Inbred Lines Across Treatments

Finally, we used the AMMI method with two principal components to identify stable ILs with their adaptation behavior in a graphical manner. Stability refers to the ability of the genotype to perform consistently, both with high or low yield levels in multiple environments, while adaptability refers to a genotype that produces high yields in specific environmental conditions and poor yields in another environment (Annicchiarico, 2002). Several ILs (ILs 6, 7, 13, 15, 18, and 19) can be regarded as stable. Both, commercial and public lines are present in this group. In general, obtained results for W treatment to a large extent correspond to the classification of used ILs to tolerant and drought-sensitive based on the 2-year identification results (ILs 9-20) or our knowledge (ILs 1-8). Namely, 10 out of 15 ILs previously determined as drought tolerant express uniform superiority in both drought and non-stress conditions (group A) or had a relatively higher biomass yield only under stress (group C). Furthermore, two public ILs known to be drought susceptible B73 (exhibits top-fire) and Mo-17 (barrenness under drought) (Chang et al., 2018) had good performance only in control (C) and not under drought condition (W) or express poor biomass performance in both drought and non-stress conditions, respectively. Three ILs (9, 1, and 4) appeared to be most adapted to mild stress (N), as their yield was high (only) under nitrogen stress but poor under water stress or under combination of nitrogen and water stress. Inbreds 9 and 1 are public, while IL 4 represents a prolific (multi-ear) version of the B73 inbred (IL1). A prolific version of B73 with higher average productivity than its original, has previously been found to be drought sensitive (Chen et al., 2012). In recent study with 98 Expired Plant Variety Protection Act-certified germplasm for genetic diversity of nitrogen use traits B73 exhibited high nitrogen use and utilization efficiency (Mastrodomenico et al., 2018). On the other hand, Mo-17 showed low physiological efficiency of plants to produce grain utilizing the plant N accumulated when grown without N fertilizer. This is well in accordance with our study since B73 and Mo-17 were classified in group A (most adapted to nitrogen stress) and group D (most sensitive to nitrogen stress), respectively. Furthermore, these two inbreds have different response to drought and nitrogen stress and combined well sa a hybrid (Hallauer et al., 2010). In general, the intermated B73-Mo17 recombinat ILs with high B73 composition had better performance under low nitrogen stress than the ones with high Mo-17 composition (Sen et al., 2015). Most adapted to severe stress conditions are ILs 10, 8, and 2, all from the public domain. They were classified in group C in all treatments (C–C–C). However, it can be noted that some of the ILs (e.g., 12 and 15) with high biomass yields only under stress treatments (C–C–C) were rather grouped among stable ILs than among ILs adapted to severe stress. This could be due to a low discriminatory power of the method (Thiry et al., 2016) to distinguish genotypes from the A and C groups and it has been suggested that genotypes from both A and C groups should be considered for selection for abiotic stress tolerance and suitable yield performance. In this regard it will be important to investigate how well the categorization of the inbreds achieved in this study using the phenotyping experiments in the controlled environmental condition matches with their classification based on field data (i.e., to assess the value of the controlled environment phenotyping data to identify lines performing well in stress conditions in the field). Thus, future work will involve the use of data from ongoing field trials to investigate the value of high-throughput image-derived traits collected and analyzed as described in this study.

## Conclusion

High-throughput plant phenotyping becomes more and more widely used in plant breeding. Image analyses provide the opportunity to study new traits which we weren’t able to measure manually, but with challenge to recognize most realible and useful biological traits. In this study, we identified several color-related traits and kinetic chlorophyll fluorescence (PSII) that might be relevant features informative of biomass production ability in maize, particularly under severe stress conditions. In addition, architectural traits related to a greater leaf area were found to provide good discrimination of resistant cultivars to abiotic stresses which are assumed to better perform under climate change scenarios. Our future work is planned to be on translating the results and predicting the effect of traits obtained from experiments under controlled greenhouse conditions to field environments.

## Author Contributions

DD, SB, JV, DI-M, and ND conceived and designed the study. DD, SB, AN, KW-F, and AJ performed the experiment. DD, SB, and MZ analyzed the data and wrote the manuscript, with contribution from AN, KW-F, and TA. AJ and TA supervised the project. All authors read and approved the final version of the manuscript.

## Conflict of Interest Statement

The authors declare that the research was conducted in the absence of any commercial or financial relationships that could be construed as a potential conflict of interest.
